# Healthy store programs and the Special Supplemental Nutrition Program for Women, Infants, and Children (WIC), but not the Supplemental Nutrition Assistance Program (SNAP), are associated with corner store healthfulness^☆^

**DOI:** 10.1016/j.pmedr.2016.06.018

**Published:** 2016-06-29

**Authors:** Robin S. DeWeese, Michael Todd, Allison Karpyn, Michael J. Yedidia, Michelle Kennedy, Meg Bruening, Christopher M. Wharton, Punam Ohri-Vachaspati

**Affiliations:** a Arizona State University, School of Nutrition and Health Promotion, 500 N 3rd St, Phoenix, AZ 85004-0698, USA; b Arizona State University, College of Nursing and Health Innovation, 500 N 3rd St, Phoenix, AZ 85004-0690, USA; c University of Delaware, Center for Research in Education, and Social Policy, 16 W Main St, Newark, DE 19716, USA; d Rutgers University, Center for State Health Policy, 112 Paterson, New Brunswick, NJ 08901-1293, USA

**Keywords:** Diet, Food, And nutrition, Nutrition surveys, Food environment, Food assistance

## Abstract

In response to lack of access to healthy foods, many low-income communities are instituting local healthy corner store programs. Some stores also participate in the United States Department of Agriculture's Special Supplemental Nutrition Program for Women, Infants, and Children (WIC) and the Supplemental Nutrition Assistance Program (SNAP). This study used two assessment tools to compare the healthfulness of offerings at stores participating in local healthy store programs (upgraded stores), WIC, and/or SNAP to that of similar non-participating stores.

Based on store audits conducted in 315 New Jersey corner stores in 2014, we calculated healthy food availability scores using subsections of the Nutrition Environment Measures Survey for Corner Stores (NEMS-CS-Availability) and a short-form corner store audit tool (SCAT). We used multivariable regression to examine associations between program participation and scores on both instruments.

Adjusting for store and block group characteristics, stores participating in a local healthy store program had significantly higher SCAT scores than did non-participating stores (upgraded: *M* = 3.18, 95% CI 2.65–3.71; non-upgraded: *M* = 2.52, 95% CI 2.32–2.73); scores on the NEMS-CS-Availability did not differ (upgraded: *M* = 12.8, 95% CI 11.6–14.1; non-upgraded: *M* = 12.5, 95% CI 12.0–13.0). WIC-participating stores had significantly higher scores compared to non-participating stores on both tools. Stores participating in SNAP only (and not in WIC) scored significantly lower on both instruments compared to non-SNAP stores.

WIC-participating and non-SNAP corner stores had higher healthfulness scores on both assessment tools. Upgraded stores had higher healthfulness scores compared to non-upgraded stores on the SCAT.

## Highlights

• Healthy corner store upgrades may increase stores' healthfulness.
• Corner stores that are WIC vendors are healthier than are non-WIC stores.
• Corner stores that are SNAP vendors are less healthy compared to non-SNAP vendors.

## 1 Introduction

Efforts to combat the rise in obesity rates in the US have resulted in a close examination of the role of the food environment, including the availability of healthy foods across localities (Escaron et al., 2013, Gittelsohn et al., 2014, Centers for Disease Control and Prevention, 2015, Larson et al., 2013, Ohri-Vachaspati et al., 2013, Rimkus et al., 2015, Zenk et al., 2014). Low-income and minority residents often suffer from obesity at higher rates than do higher income, non-minority residents, and as such, consideration of the food environment as it pertains to these higher risk groups is a priority. Recent data show that low-income and high-minority communities have an abundance of small retail food stores such as convenience and corner stores, but frequently lack supermarkets (Powell et al., 2007, Moore and Roux, 2006). Corner stores stock a greater proportion of energy-dense, nutrient-poor foods, combined with fewer fresh fruits and vegetables (FV), whole grains, and low-fat dairy than do supermarkets (Laska et al., 2010, Borradaile et al., 2009). As a result, low-income, high-minority neighborhoods often have limited access to healthy foods.

In response to these disparities, many communities have instituted healthy corner store programs that encourage and support healthy upgrades to corner stores. As interventions in small stores proliferate, early results, some assessed by validated, comprehensive store audits (Cavanaugh et al., 2014, Paek et al., 2014) and some assessed by study-specific measurement tools,(Song et al., 2009, Dannefer et al., 2012, Ayala et al., 2013) demonstrate good success at increasing the availability of healthy foods (Cavanaugh et al., 2014, Paek et al., 2014, Song et al., 2009, Dannefer et al., 2012, Ayala et al., 2013). Only a few evaluations, however, have compared corner stores involved in healthy initiatives to stores that are not (Song et al., 2009, Ayala et al., 2013).

Federal programs seek to provide additional food purchasing assistance to low-income families, but have been the source of ongoing debate and scrutiny. The Special Supplemental Nutrition Program for Women, Infants, and Children (WIC), for example, provides vouchers for specific designated products based on their nutrient profile. Stores that accept WIC vouchers must stock a variety of healthy foods including reduced-, low-, or non-fat milk; 100% unsweetened juice; FV sold fresh, canned (in water or their own juice and with no added sodium), frozen (with no added sugars or sodium), or dried; and whole grains (USDA Food and Nutrition Service, 2014).

In contrast, the Supplemental Nutrition Assistance Program (SNAP) provides a source of funding for the purchase of almost any food or beverage with few exceptions. SNAP vendors must sell at least three varieties of foods in four staple food groups: meat, poultry, or fish; bread or cereal; vegetables or fruits; and dairy products. Nutrient requirements are currently not in place for SNAP-authorized foods. Grains are not required to be whole, fat content is not specified for any foods, and canned and frozen FV have no sugar or sodium restrictions (USDA Food and Nutrition Service, 2013).

Stores are required to receive state-administered certification as eligible vendors for these programs. Nationally, SNAP vendors outnumber WIC vendors 5:1 (USDA Supplemental Nutrition Assistance Program, 2014, USDA Food and Nutrition Service, Office of Policy Support,). Corner stores that accept vouchers for WIC have been shown to stock a greater number of healthy foods compared to non-WIC stores (Tester et al., 2011), likely due to the federally-mandated stocking requirements for WIC vendors.

This study compared the availability of healthy foods in corner stores participating in WIC, SNAP, and/or a healthy corner store program to that of non-participating similar stores across four cities (Camden, Newark, New Brunswick, and Trenton) in New Jersey. Two related but distinct instruments were used to examine these differences.

## 2 Methods

The study design, sampling approach, and development of a reduced corner store audit instrument have been described previously (DeWeese et al., in press).

### 2.1 Audit instruments

Assessments of stores' healthy food offerings were made using subsections of an existing comprehensive audit tool, the Nutrition Environment Measures Survey for Corner Stores (Cavanaugh et al., 2013) (NEMS-CS) and the newly developed short-form corner store audit tool (SCAT) (DeWeese, Todd, Karpyn, Yedidia, Kennedy, Bruening, Wharton, Ohri-Vachaspati, 2016, unpublished data under review). The full NEMS-CS is intended for in-person appraisals of availability, quality, and prices of foods from 13 different categories (milk, fruit, frozen and canned fruits, vegetables, frozen and canned vegetables, ground beef, hot dogs, frozen dinners, baked goods, beverages, bread, baked chips and snacks, and cereal). Because one aim of the original project was to test the feasibility of administering the reduced audit over the phone, the NEMS-CS version used in the current study was constructed by retaining only items assessing availability of healthy foods, while NEMS-CS items related to price and quality, which are difficult to administer reliably over the telephone, were excluded. The version used in this study is referred to as NEMS-CS-Availability.

The SCAT is a validated instrument (Pearson correlation of 0.79 between SCAT and NEMS-CS availability scores), developed using the NEMS-CS-Availability tool and store audits (DeWeese et al., in press). It requires fewer resources compared to comprehensive store audit tools to capture corner stores' healthfulness levels. Whereas the NEMS-CS measures the availability of over 50 individual items, the SCAT, a seven-item instrument, measures the availability of skim/1% milk, fresh fruits (five or more types vs four or fewer), fresh vegetables (five or more types vs four or fewer), frozen vegetables, and ground meat, as well as the presence of WIC signage and refrigeration for meat, fruits, or vegetables.

### 2.2 Sample and procedures

Corner store audits were conducted from June through December 2014 in 325 stores using the NEMS-CS-Availability and the SCAT. Auditors were trained and independently conducted practice audits in pairs to determine inter-rater reliability. Four items had kappa values under 0.7 and these were clarified during further training. Two independent auditors completed the audits in each store.

The sample size was based on simulation studies examining required sample sizes for exploratory factor analysis (Mundfrom et al., 2005), which was used in development of the SCAT. The sampling frame for data collection consisted of the 781 small food stores listed in 2013 InfoUSA and Nielsen commercial databases for the metro areas of Camden, Newark, Trenton, and New Brunswick, New Jersey, communities that are part of the New Jersey Child Health Study (NJCHS). These cities have received funding from various sources including, but not limited to, the Robert Wood Johnson Foundation and the American Heart Association to produce and support policy and environmental changes to increase access to healthy foods (Robert Wood Johnson Foundation, 2015). One strategy adopted by some community partners was to work with local small food retailers to upgrade their stores to stock and promote healthier options (Change Lab Solutions, 2014). A number of organizations, including The Food Trust (The Food Trust, 2012) and the New Jersey Partnership for Healthy Kids (Robert Wood Johnson Foundation, 2015), facilitate the corner store upgrades in these communities. The organizations work closely with the NJCHS, continually providing updates on their involvement in the healthy corner store programs. At the initiation of store audits 43 stores in the study areas were participating in upgrade programs. These 43 upgraded stores were included in the sample, in addition to 282 stores that were randomly selected from the pool of all non-upgraded stores in the four cities. Stores in which employees refused audits (n = 2), that could not be located in the field (n = 7), or that were found to be permanently closed (n = 19) were replaced in the sample by corner stores observed in close proximity (usually within a block) to the original store. This study did not involve human subjects and was therefore granted an exemption from review by the Arizona State University Institutional Review Board.

### 2.3 Audit instrument scoring

NEMS-CS-Availability audit scores were calculated using the product availability portion of the full NEMS-CS scoring algorithm (Center for Health Behavior Research - University of Pennsylvania, 2014). Product availability is scored on a scale of 0–34, and is calculated by adding scores from each of the 13 categories. While some items are scored solely on whether or not they are present, others are assigned differential weights based on their nutritional value (e.g., higher score for low-fat vs. whole milk) or on the number of varieties available. The higher the score, the more healthy items were observed during in-store audits. Scores on the SCAT were calculated by assigning one point for the presence of an item/signage, for a total possible score of seven points; scores were based on in-store observations of the seven SCAT items.

### 2.4 Store characteristics

Data on stores' total sales volume and size in square feet were obtained from InfoUSA and Nielsen databases. The organizations involved in executing the healthy store programs, including The Food Trust (The Food Trust, 2012) and the New Jersey Partnership for Healthy Kids (Robert Wood Johnson Foundation, 2015), provided the information about which study area stores were enrolled in healthy upgrade programs. Information on stores' WIC and/or SNAP participation was obtained by observing the presence or absence of WIC and/or SNAP signs during in-store audits. Stores were defined as 1) upgraded versus non-upgraded, 2) WIC vendors versus non-WIC vendors (regardless of whether they were also SNAP vendors), and 3) SNAP vendors versus non-SNAP vendors (excluding any that were also WIC vendors). Categories were not mutually exclusive (e.g., an upgraded store could also be a WIC vendor).

### 2.5 Neighborhood characteristics

Addresses of all stores were geocoded using ArcGIS. Store names and addresses were matched with their corresponding Census block group's demographic characteristics, including race/ethnicity, ratio of income to poverty level, and education level. Block groups were defined by the group that composed at least a 51% proportion of a given characteristic. For example, block groups in which 51% or more of residents had only a high school education or less were defined as “majority high school education or less.” Block group characteristics were obtained from the US Census' American Community Survey (US Census Bureau, 2013).

### 2.6 Statistical analysis

Frequencies were used to summarize categorical variables. Independent t-tests were conducted to compare mean proportions of households earning < 150% of the Federal Poverty Level (FPL) in block groups in which upgraded versus non-upgraded stores, WIC-certified versus non-WIC-certified stores, and SNAP-certified versus non-SNAP-certified stores were located. Multivariable regression analyses were used to examine associations between scores on both the NEMS-CS-Availability and SCAT and 1) upgraded store status, 2) WIC vendor status, 3) SNAP-only vendor status. Interaction effects of WIC x upgraded stores and SNAP x upgraded stores on audit scores were also explored to examine whether any observed associations between scores and a store's status as upgraded could be attributed to the store's WIC or SNAP certification status. All models adjusted for stores' sales volume and size (in square feet), as well as the majority education level (high school or less vs. all others), majority race/ethnicity (non-Hispanic black, Hispanic vs. non-Hispanic white, mixed), and majority income level (< 150% of FPL vs. ≥ 150% of FPL) of the block groups in which stores were located. Adjusted means for NEMS-CS-Availability and SCAT scores were calculated from the regression model results using the margins command in Stata (version 12.1, 2011, StataCorp).

Overall, 10 of the audited stores could not be found in the InfoUSA or Nielsen databases and were therefore dropped from the analysis. Four of these 10 stores were on the list of upgraded stores, and the remaining six were stores selected as replacements for permanently closed stores. One other store was dropped because it had missing information from the in-store audit, which precluded calculation of a NEMS-CS-Availability score for that store. Thus, n = 314 stores were available for multivariable analyses using the NEMS-CS-Availability and n = 315 stores for analyses using the SCAT (n = 39 upgraded stores in both samples). Analyses were conducted in 2014 and 2015.

## 3 Results

**Fig. 1:**
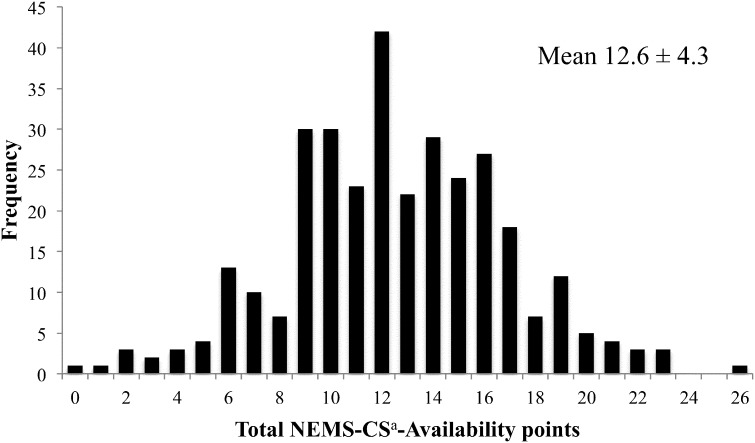
Distribution of NEMS-CS^a^-Availability scores obtained from 314 corner store audits in 2014 in four New Jersey cities ^a^Nutrition Environment Measures Survey for Corner Stores.

**Fig. 2:**
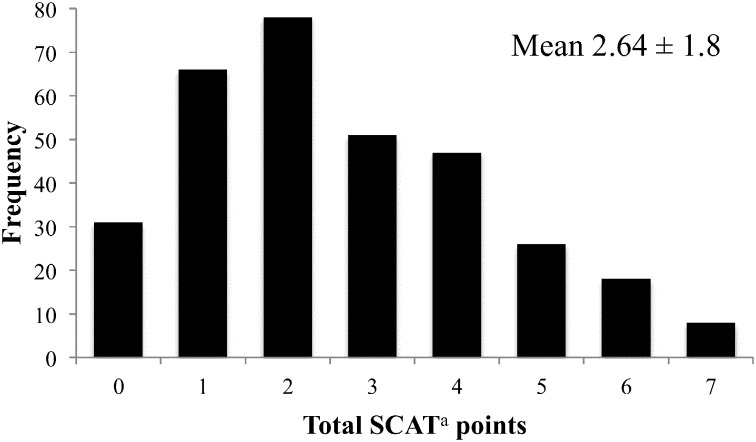
Distribution of SCAT^a^ scores obtained from 315 corner store audits in 2014 in four New Jersey cities ^a^Short form Corner store Audit Tool.

Observed scores on the NEMS-CS-Availability audit ranged from 0 to 26, with a mean score of 12.6 ± 4.3 (Fig. 1). SCAT audit scores ranged from 0 to 7, with a mean score of 2.64 ± 1.8 (Fig. 2). In 91% of block groups, the majority of residents had at least a high school education or high school equivalency diploma (Table 1). Eighty-three percent of block groups were majority black or Hispanic. Approximately 80% of stores were 1250 square feet or smaller, and most had sales volumes less than a million dollars.

A greater proportion of households in block groups in which WIC-certified stores were located earned < 150% of the FPL compared to block groups in which stores that did not accept WIC were located (0.49 ± 0.02 vs 0.43 ± 0.01, p < 0.05). After adjusting for store and other block group characteristics, on average, stores located in communities in which the majority of households earned < 150% of the FPL had significantly higher NEMS-CS-Availability scores compared to stores in communities in which the majority of households earned > 150% of the FPL (*M* = 13.2, 95% CI 12.4–14.0 vs *M* = 12.1, 95% CI 11.6–12.7).

On average, upgraded stores had significantly higher SCAT scores (*M* = 3.18, 95% CI 2.65–3.71) compared to non-upgraded stores (*M* = 2.52, 95% CI 2.32–2.73) after adjusting for store and block group characteristics (Table 2). Upgraded versus non-upgraded store status was not associated with NEMS-CS-Availability scores (upgraded: *M* = 12.8, 95% CI 11.6–14.1; non-upgraded: *M* = 12.5, 95% CI 12.0–13.0). WIC vendors had significantly higher scores compared to non-WIC vendors on both the NEMS-CS-Availability (WIC: *M* = 15.3, 95% CI 14.4–16.1; non-WIC: *M* = 11.6, 95% CI 11.1–12.1) and the SCAT (WIC: *M* = 4.29, 95% CI 3.98–4.60; non-WIC: *M* = 2.01, 95% CI 1.83–2.20). SNAP-only vendors scored significantly lower on both instruments (NEMS-CS-Availability: *M* = 11.5, 95% CI 10.8–12.2; SCAT: *M* = 1.98, 95% CI 1.70–2.27) compared to non-SNAP vendors (NEMS-CS-Availability: *M* = 13.2, 95% CI 12.6–13.8; SCAT: *M* = 3.04, 95% CI 2.80–3.28). Interaction effects were not observed for either WIC by upgraded stores or SNAP by upgraded stores (data not shown).

## 4 Discussion

The present study is unique in that it uses two different, but related, metrics to assess the associations between various store characteristics and healthy food availability scores. Most notable is the difference in how well the NEMS-CS-Availability and the SCAT discriminated between upgraded versus non-upgraded stores. SCAT scores were 26% higher for upgraded stores compared to non-upgraded stores, suggesting the shorter tool is effective at detecting differences in healthy food availability when efforts to improve the store environment are in place. Interestingly, the seven items included in the SCAT coincide closely with items included as part of minimum stocking guidelines for small retail food stores, recently created by an expert panel for Robert Wood Johnson's Healthy Eating Research program (Laska & Pelletier, 2016).

This study is the first to use both a comprehensive and a brief audit tool to compare intervention to control stores. Although the SCAT was more effective than the NEMS-CS-Availability at distinguishing intervention from control stores, in the absence of other studies using control stores rather than pre- post- interventions, it is unclear whether a short instrument would consistently be better at distinguishing between upgraded versus non-upgraded stores. Nonetheless, it may be reasonable to assume that overall scores on a tool assessing only items that are the focus of healthy upgrades will be impacted to a greater degree than will overall scores on a tool in which the items may comprise only a small proportion of the overall instrument.

Consequently, the lack of difference in NEMS-CS-Availability scores between upgraded versus non-upgraded stores is likely due to the quantity of items included in the NEMS-CS-Availability. Whereas the NEMS-CS-Availability included over 50 items in 13 categories, the SCAT included only seven items, which are generally items of focus during healthy upgrades. Few studies have compared upgraded stores to control stores to assess the effectiveness of the upgrade at increasing the availability of healthy foods. Rather, most have conducted pre- post-analysis, assessing the healthfulness score before a healthy upgrade, and again after upgrades have been initiated (Cavanaugh et al., 2014, Paek et al., 2014). Two initiatives that used control stores were the Baltimore Healthy Stores (BHS) (Song et al., 2009) and the Vida Sana: Hoy y Mañana tienda-based interventions (Ayala et al., 2013). The BHS focused on increasing the availability of only 10 items, and Vida Sana: Hoy y Mañana focused on increasing fruits and vegetables. As could be expected, availability scores, which measured only the items of focus, similar to the SCAT in the current study, were higher among intervention compared to control stores in both studies.

This is the first study to look at the availability of healthy foods in corner stores participating in three programs – SNAP, WIC, and healthy corner store programs – on a multi-city scale within a state. While all three programs have been examined closely in recent years as mechanisms to improve healthy food purchasing behaviors, no other systematic multi-city-level evidence of the comparative impact of the programs on increasing healthy food availability has been observed.

As other studies have shown (Tester et al., 2011, Havens et al., 2012, Hillier et al., 2012, Rose et al., 2014), WIC vendors had significantly higher scores compared to non-WIC vendors on both the SCAT and the NEMS-CS-Availability. Although four of the six food items included in the SCAT are WIC-authorized foods, a significant interaction between upgraded stores and WIC certification was not observed, indicating that higher SCAT scores were associated with upgraded stores regardless of whether or not the stores were WIC vendors.

SNAP vendor status was associated with lower scores on both instruments, contrasting sharply with the WIC program in the current study when considering that WIC-authorized stores provide greater healthy food availability to all neighborhood residents, regardless of residents' status as WIC-recipients. SNAP-authorized stores, on the other hand, do not improve healthy food availability to neighborhood residents. Current SNAP regulations may partially explain the observed results. While SNAP vendors must offer at least three varieties of items in each of four staple food groups, current regulations do not explicitly prohibit prepared mixtures with multiple ingredients (e.g., cold pizza or macaroni and cheese) or accessory food items such as chips and crackers from being included as part of staple food groups. A proposed rule by the Food and Nutrition Service would more strictly define staple foods, as well as require perishable foods to be available in three of the staple categories (USDA Food and Nutrition Service, 2016). These changes could result in increased healthy food access for communities in which SNAP-certified vendors are located. Martin et al. (Martin et al., 2012) observed 12% and 15% greater odds of customers purchasing fruits and vegetables, respectively, for each additional type of fruit or vegetable stocked in corner stores. Further, compared to non-SNAP recipients, SNAP customers were 1.7 times more likely to purchase fruits. SNAP holds great, untapped potential for increasing healthy food availability in low-income areas.

It must be noted that simply making healthy foods available will not necessarily result in consumers purchasing those items. Hoffman et al. (Hoffman et al., 2009) and Lent et al. (Lent et al., 2014) incentivized youth to purchase healthier items from corner stores, but the 2-year interventions did not impact purchasing behaviors. However, public health advocates have a responsibility to pursue and promote approaches to decrease healthy food disparities that result from the lack of supermarkets in low-income, high-minority communities, in order to provide low-income residents with the same option as residents of high-income neighborhoods have to purchase healthy foods if they choose.

Study limitations included the relatively small sample size of upgraded stores; however, confidence intervals were fairly precise. Additionally, when the list of stores in which window and/or shelf signs indicating WIC acceptance were compared with the list of authorized WIC vendors supplied by the New Jersey Department of Health (NJDOH) (State of New Jersey Department of Health, 2015), almost half of stores in which signage was observed were not found on the NJDOH list, likely due to WIC vendor authorization expiring every three years. In sensitivity analysis, results with both instruments were almost identical using either the in-store signage list or the NJDOH list, indicating that previous certification as a WIC vendor had a continuing association with a store's healthfulness. A study strength was controlling for store size and sales volume and the block group characteristics most likely to affect store inventory.

## 5 Conclusions

These results demonstrate that WIC and non-SNAP corner stores are associated with higher healthfulness scores using the NEMS-CS-Availability and SCAT instruments, and that healthy upgrades to corner stores seem to be effective. Further research should be conducted to more comprehensively study the effects of store upgrades in relation to participation in federal food programs and other healthy initiatives.

## Funding

This work was funded by a National Institute of Food and Agriculture predoctoral dissertation fellowship (grant number 20146701122279) and by a grant from the National Institute of Child Health and Human Development (grant number 1R01HD071583-01A1).

## Conflict of interest statement

The authors declare that there are no conflicts of interest.

## Figures and Tables

**Table 1:** Block group and store characteristics of New Jersey corner stores in which audits of their product availability were conducted in 2014.

	n	%
**Majority characteristic of block group (n = 325)**		
Mixed race	54	16.6
Hispanic	117	36.0
Black	154	47.4
< 150% FPL^a^	117	36.0
At least HS^b^ education/HS^b^ equivalency	295	90.8
**Store program participation (n = 325)**		
Upgraded stores	43	13.2
WIC^c^ vendors	88	27.1
SNAP^d^ only vendors	133	40.9
**Store characteristics (n = 315)**		
Sales volume ($)		
< 500,000	30	9.5
≥ 500,000–<1,000,000	256	81.3
≥ 1,000,000	29	9.2
Size (feet^2^)		
≤ 1250	296	94.0
> 1250	19	6.0

a Federal poverty level.

b High school.

c Special Supplemental Nutrition Program for Women, Infants, and Children.

d Supplemental Nutrition Assistance Program (excludes any stores that were also WIC vendors).

**Table 2:** Adjusted mean scores on NEMS-CS^a^-Availability and SCAT^b^ instruments by store program participation in New Jersey corner stores in which product availability audits were conducted^c^ in 2014.

	Marginal means (95% CI)	
NEMS-CS^a^-availability points (n = 314)	SCAT^b^ points (n = 315)
Upgraded^d^	12.8 (11.6–14.1)	3.18 (2.65–3.71)⁎
Non-upgraded	12.5 (12.0–13.0)	2.52 (2.32–2.73)
WIC^e^ vendors	15.3 (14.4–16.1)⁎	4.29 (3.98–4.60)⁎
Non-WIC^e^ vendors	11.6 (11.1–12.1)	2.01 (1.83–2.20)
SNAP^f^-only vendors	11.5 (10.8–12.2)⁎	1.98 (1.70–2.27)⁎
Non-SNAP^f^-only vendors	13.2 (12.6–13.8)	3.04 (2.80–3.28)

a Nutrition Environment Measures Survey for Corner Stores.

b Short form Corner store Audit Tool.

c Separate multivariable regression models run for each. All models adjusted for stores' sales volume and size; block group: majority education level, majority race/ethnicity, majority income level. Upgraded × WIC and upgraded × SNAP interactions were tested but not observed.

d Does not exclude WIC and SNAP vendors.

e Special Supplemental Nutrition Program for Women, Infants, and Children.

f Supplemental Nutrition Assistance Program (excludes any stores that were also WIC vendors).

⁎ *p* < 0.05 for differences between upgraded vs non-upgraded, WIC vs non-WIC, and SNAP-only vs non-SNAP-only.
